# Core genome multilocus sequence typing of *Clostridioides difficile* to investigate transmission in the hospital setting

**DOI:** 10.1007/s10096-023-04676-9

**Published:** 2023-10-23

**Authors:** Paraskevas Filippidis, Laurence Senn, Fabrice Poncet, Bruno Grandbastien, Guy Prod’hom, Gilbert Greub, Benoit Guery, Dominique S. Blanc

**Affiliations:** 1 Infection Prevention and Control Unit, Infectious Diseases Service, Lausanne University Hospital and University of Lausanne, Lausanne, Switzerland; 2 Infectious Diseases Service, Lausanne University Hospital and University of Lausanne, Lausanne, Switzerland; 3 Swiss National Reference Center for Emerging Antibiotic Resistance (NARA), University of Fribourg, Fribourg, Switzerland; 4 Institute of Microbiology, Lausanne University Hospital and University of Lausanne, Lausanne, Switzerland

**Keywords:** *Clostridioides difficile*, Bacterial genomics, Epidemiology, Molecular typing, Outbreak investigation, cgMLST

## Abstract

**Purpose:**

Traditional epidemiological investigations of healthcare-associated *Clostridioides difficile* infection (HA-CDI) are often insufficient. This study aimed to evaluate a procedure that includes secondary isolation and genomic typing of single toxigenic colonies using core genome multilocus sequence typing (cgMLST) for the investigation of *C. difficile* transmission.

**Methods:**

We analyzed retrospectively all toxigenic *C. difficile*-positive stool samples stored at the Lausanne University Hospital over 6 consecutive months. All isolates were initially typed and classified using a modified double-locus sequence typing (DLST) method. Genome comparison of isolates with the same DLST and clustering were subsequently performed using cgMLST. The electronic administrative records of patients with CDI were investigated for spatiotemporal epidemiological links supporting hospital transmission. A comparative descriptive analysis between genomic and epidemiological data was then performed.

**Results:**

From January to June 2021, 86 *C. difficile* isolates were recovered from thawed samples of 71 patients. Thirteen different DLST types were shared by > 1 patient, and 13 were observed in single patients. A genomic cluster was defined as a set of isolates from different patients with ≤ 3 locus differences, determined by cgMLST. Seven genomic clusters were identified, among which plausible epidemiological links were identified in only 4/7 clusters.

**Conclusion:**

Among clusters determined by cgMLST analysis, roughly 40% included unexplained HA-CDI acquisitions, which may be explained by unidentified epidemiological links, asymptomatic colonization, and/or shared common community reservoirs. The use of DLST, followed by whole genome sequencing analysis, is a promising and cost-effective stepwise approach for the investigation of CDI transmission in the hospital setting.

**Supplementary Information:**

The online version contains supplementary material available at 10.1007/s10096-023-04676-9.

## Introduction

*Clostridioides difficile* is a strict anaerobe, spore-forming, Gram-positive bacillus and a leading cause of healthcare-associated diarrhea, responsible for 15–30% of post-antibiotic diarrhea in humans [1]. Between 4 and 15% of the general adult population is colonized by *C. difficile*, among which 6–70% are toxigenic strains [2]. Toxigenic *C. difficile* produces three toxins, namely the binary toxin (CDT), toxin A (TcdA), and toxin B (TcdB), which are the main virulence factors and are coded by the *Cdt* locus and the *tcdA* and *tcdB* genes (both located on the pathogenicity locus (PathLoc)), respectively [3]. Toxin B is 100 to 1000 times more toxic to human cells than toxin A. It plays a major role in the pathogenesis of *C. difficile* infection (CDI) and has been associated with a higher incidence of severe disease, complications, and recurrence [4, 5].

Although variable incidences of CDI and heterogeneous methodologies of epidemiological surveillance have been reported worldwide, CDI has been clearly acknowledged as a substantial burden in healthcare facilities across many countries [6] and has become the focus of numerous infection control strategies [1, 7]. Despite the optimization of isolation measures and cleaning procedures, and the efforts to improve antibiotic stewardship, a major impact of CDI in the healthcare setting is still observed and underlines the need for a better understanding of its nosocomial transmission. However, traditional epidemiological investigation approaches alone are often insufficient, since patient trajectories within the hospital may be complex, and encounters with asymptomatic but possibly contagious patients are often not taken into account [8]. Hence, there is currently an unmet need for new strategies to investigate healthcare-associated CDI (HA-CDI). This requirement could be effectively tackled through contemporary genotyping techniques that offer genetic resolution, directly yielding information regarding transmission pathways and the ability to detect temporal alterations in the bacterial genome. Consequently, isolates exhibiting a high degree of genetic similarity are more likely to be associated with a recent transmission chain [8, 9]. Although PCR ribotyping is traditionally used for *C. difficile* typing, its utility in the investigation of transmission and outbreaks remains limited. By contrast, core genome multilocus sequence typing (cgMLST) has been shown to have a much higher performance in discriminating distinct bacterial strains and is a promising tool for epidemiological investigation of CDI [10]. While several techniques have been used for molecular typing of *C. difficile* to date [11], *C. difficile* stool cultures have been mostly abandoned, leading to the absence of isolates for further genomic analyses.

In our laboratory, *tcdB*-positive stool samples are systematically stored and may be used for further culturing, strain isolation, and molecular typing [12]. Our study aimed to evaluate a procedure that includes secondary isolation and genomic typing of isolated toxigenic colonies using cgMLST for the investigation of HA-CDI.

## Methods

### Setting and strain collection

Lausanne University Hospital is a 1100-bed teaching hospital which serves as a primary-level community hospital for Lausanne (catchment population of 300,000 people) and as a secondary and tertiary referral hospital for Western Switzerland (catchment population of 1–1.5 million people). CDI diagnosis is confirmed based on the IDSA/SHEA criteria of diarrhea and a positive CDI test for a toxigenic strain [13]. *C. difficile* toxins are detected in stools through nucleic acid amplification tests (GeneXpert *C. difficile*, BT, Cepheid, Sunnyvale, CA, USA) with or without a positive result for glutamate dehydrogenase (GDH) and/or enzyme immunoassay for toxins A/B. All *tcdB*-positive stool samples are systematically stored in a 1.5-ml microtube at − 80 °C for 2 years.

### Microbiology

Stored *C. difficile*-positive stool samples were thawed and inoculated onto chromID *C. difficile* medium (bioMérieux, France) using a 10-µl loop. Incubation in strict anaerobic conditions at 37 °C was subsequently performed for 24 h, as recommended by the manufacturer. At least one isolate per stool was used for further analysis, and PCR assay was performed to confirm the presence of toxin genes, as previously described [12].

### Molecular and genomic typing

All isolates were typed using a previously published modified double-locus sequence typing (DLST) scheme [14] (see supplementary material). One isolate per patient and per DLST type was further sequenced using the Illumina MiSeq platform. Sequence reads were analyzed using BioNumerics™ (version 8.1, available at http://www.applied-maths.com) with default settings, except the de novo assembly, which was performed using the Unicycler pipeline. MLST was determined with the public MLST scheme available at https://pubmlst.org/organisms/clostridioides-difficile. For genome comparison, we performed cgMLST using a scheme developed by Applied Maths. Clustering was performed using the categorical-difference coefficient, and a minimum spanning tree for categorical data was built with single- and double-locus variance priority rules. Based on previous publications [15, 16], a genomic cluster was defined as the set of isolates from different patients with differences of 3 loci or less [10, 17–21]. All sequence read files have been deposited in the European Nucleotide Archive database under the study project number PRJEB56399.

### Epidemiological investigation and definitions

The electronic administrative records of patients diagnosed with CDI were investigated for epidemiological links supporting hospital transmission including shared space and for how long (at the room or ward level), as well as overlapping hospital stays. HA-CDI was defined as CDI diagnosed (positive test on unformed stool specimen) on or after hospital day 4, and community-associated CDI (CA-CDI) was defined as CDI diagnosed prior to hospital day 4. Three levels of epidemiological transmission links were defined: type A link (patient-to-patient) was considered when CDI cases were hospitalized in the same room or in the same ward at the same time, type B (ward-to-patient) when CDI cases were hospitalized in the same ward but at different times with the presumed secondary case being hospitalized within 30 days after discharge of the presumed index case, and type C when CDI cases had overlapping hospital stays in different wards. Type A link was considered the strongest and type C link the weakest.

## Results

**Fig. 1:**
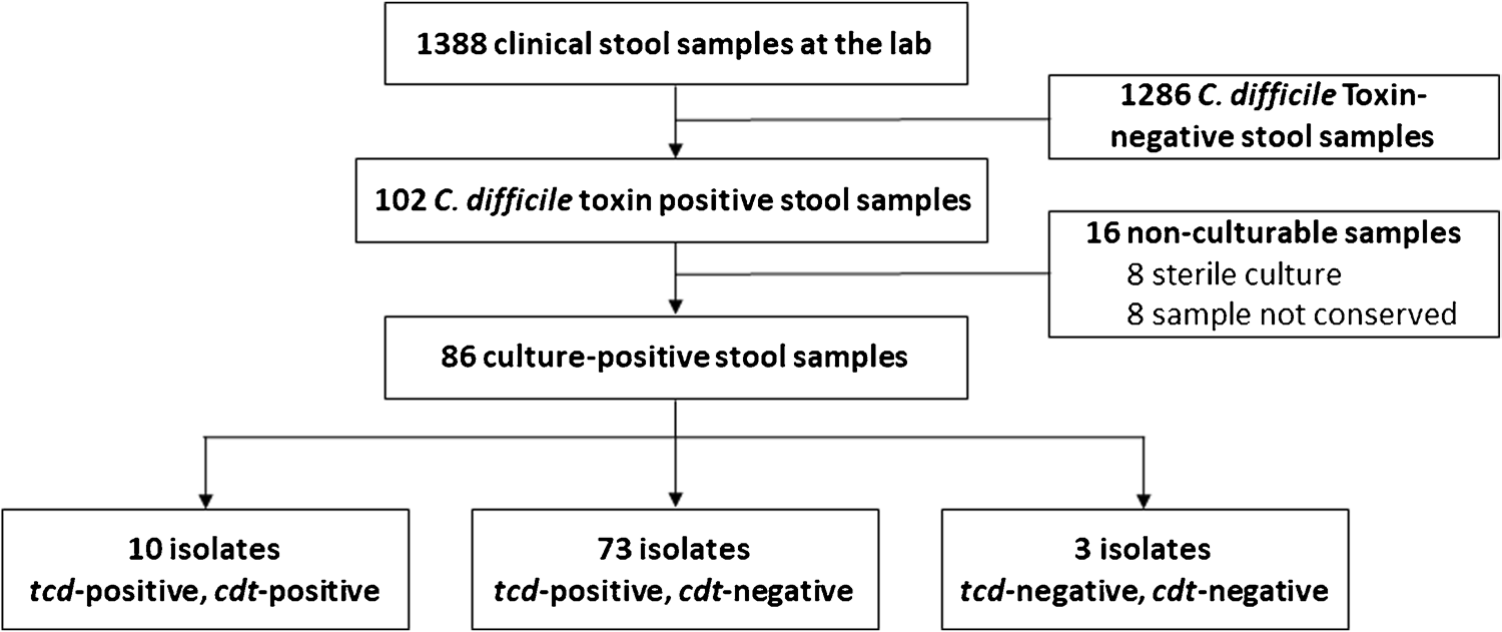
Flow diagram of included isolates. *tcd*, locus containing toxin A/B-coding genes; *cdt*, locus containing binary toxin-coding genes

From January to June 2021, 102 out of 1388 (7.3%) clinical stool samples analyzed for *C. difficile* toxin were positive and thus stored frozen. Among these, we cultured *C. difficile* from 86 thawed samples (84%) collected from 71 different patients (Table S1). Regarding the 16 remaining samples, 8 yielded no growth of *C. difficile* and 8 had not been stored. Among the 86 positive cultures, 10 were positive for all three toxin genes (*tcd*-positive, *cdt*-positive), 73 were positive for toxin A/B genes and negative for binary toxin gene (*tcd*-positive, *cdt*-negative), and three were negative for all toxin genes (*tcd*-negative, *cdt*-negative) (Fig. 1).

### Genotypic investigation

The 86 isolates belonged to 26 DLST sequence types. Thirteen different DLST were observed in 13 single patients, suggesting no transmission between them and other patients. The remaining 13 DLST types were shared within groups of two to 10 patients (total: 59 patients). Only one isolate per patient and per DLST type was further analyzed by whole genome sequencing (*n* = 72). Isolates with unique DLST were also sequenced to confirm they were genetically different from each other.

**Fig. 2:**
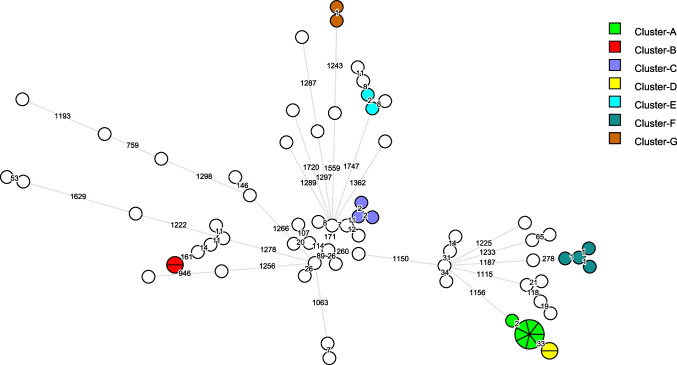
Minimum spanning tree based on cgMLST analysis of 72 *C. difficile* isolates. Each circle represents one or several isolates. The distance between circles indicates the number of different loci between the two linked isolates. When there is no locus difference between isolates, these are included in a pie circle. Genomic clusters (isolates with the same MLST and ≤ 3 different loci) are represented with different colors, and ST is provided within or near the circles

**Fig. 3:**
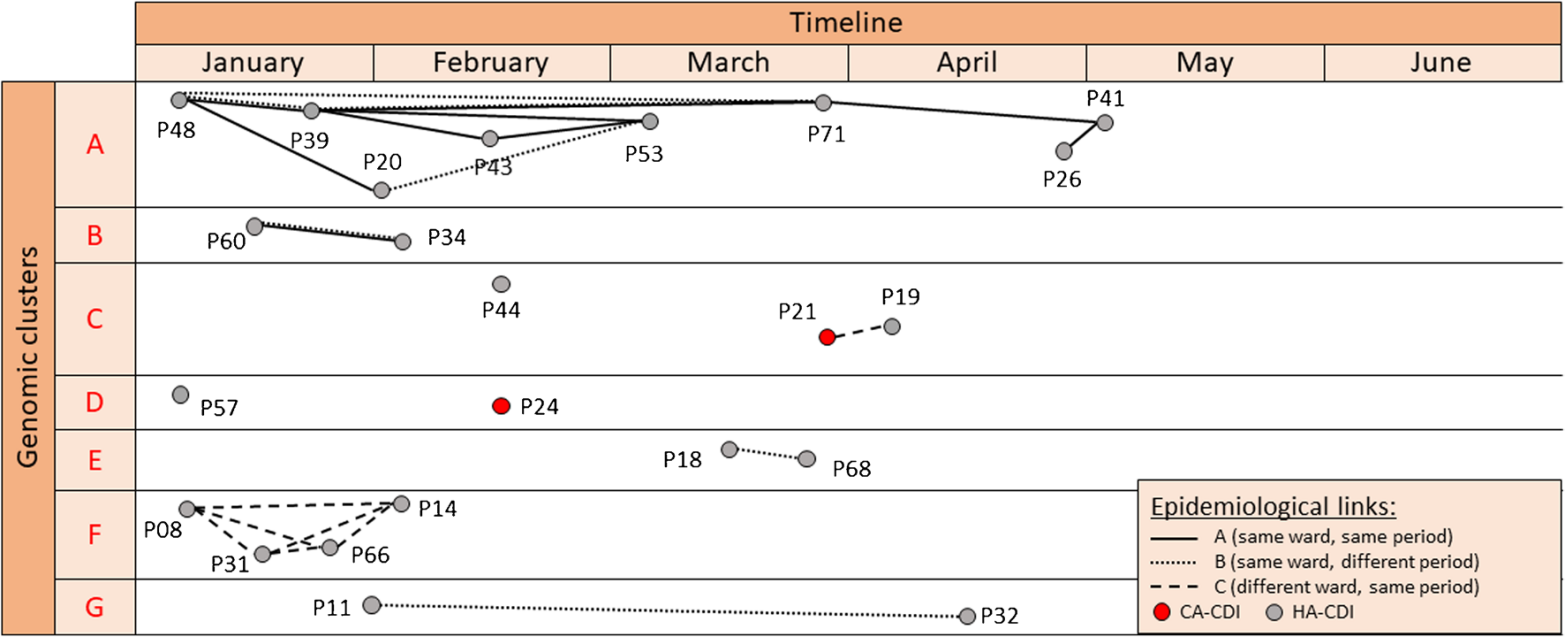
Epidemiological investigation of CDI cases within each genomic cluster across time. Clusters are represented by capital letters (A–G). Each circle represents one *C. difficile* case (red, CA-CDI; gray, HA-CDI), and each line color represents a different transmission chain. Three levels of epidemiological links were defined: type A (patient-to-patient) was considered when CDI cases were hospitalized in the same room or in the same ward at the same time, type B (ward-to-patient) when CDI cases were hospitalized in the same ward but at different times with the presumed secondary case being hospitalized within 30 days after discharge of the presumed index case, and type C when CDI cases had overlapping hospital stays in different wards. Type A link was considered the strongest and type C link the weakest

In silico analysis of the WGS data assigned the 72 isolates to 28 different MLST sequence types (ST), with ST-2 (*n* = 11 isolates) and ST-8 (*n* = 10 isolates) being the most common. Detailed data of the analyzed isolates are provided in Table S1. Analysis of cgMLST showed 1982/1992 variable loci. A cgMLST-based clustering of the isolates in the form of a minimum spanning tree was performed (Fig. 2). The higher resolution of cgMLST compared to MLST or DLST revealed that isolates within the same ST may exhibit differences of multiple loci among them (Fig. 2). Using a threshold of ≤ 3 locus differences to identify possible transmissions, seven clusters (A to G) of two to eight patients were identified (Fig. 3). Overall, 49 (68%) of 72 isolates-patients presented a unique genotype (> 3 locus differences with other isolates), suggesting transmission from or to another patient was unlikely.

### Epidemiological investigation

Among the 23 patients included in the seven clusters, 21 were classified as having HA-CDI and 2 CA-CDI (Table 1). An epidemiological link could be found in 20 (87%) of 23 patients (Fig. 3). Type A links (hospitalization in the same unit during the same period) were present in two clusters (A, *n* = 8 and B, *n* = 2), type B links (hospitalization in the same unit at different time) in two clusters (E, *n* = 2 and G, *n* = 2), type C links (hospitalization in different units at the same period) in two clusters (C, *n* = 2 and F, *n* = 4), and no link in one cluster (D, *n* = 2). One patient in cluster C had no link with the two other patients.

**Table 1:** Distribution of cases in clusters according to their origin

Cluster	Genotype	CA-CDI	HA-CDI	Total
Cluster-A	ST8-A		8	8
Cluster-B	ST54-A		2	2
Cluster-C	ST2-A	1	2	3
Cluster-D	ST8-B	1	1	2
Cluster-E	ST11-A		2	2
Cluster-F	ST34-A		4	4
Cluster-G	STnew-A		2	2

Abbreviations: *CDI*, *Clostridioides difficile* infection; *CA-CDI*, community-associated CDI; *HA-CDI*, healthcare-associated; *ST*, sequence type

## Discussion

In the present study, we investigated the molecular epidemiology of all CDI cases that occurred at the Lausanne University Hospital over a 6-month period using DLST and cgMLST. Based on previous analysis of the diversity of cgMLST in microevolution [10, 17, 18, 21], we considered isolates with a difference of 3 loci or less as more likely to be epidemiologically linked. Thus, over the study period, we identified 7 genomic clusters.

Given the potential for patient-to-patient and ward-to-patient transmission of *C. difficile*, we investigated the genetic clusters for potential epidemiological links classified in three different categories (types A, B, or C links), as described above and in previous publications [22, 23]. Among the 7 clusters determined by cgMLST analysis, four were supported by consistent epidemiological data (type A or B link).

The presence of type A links (patient-to-patient) in two clusters suggests transmission by direct contact between patients or indirect contact through healthcare workers, shared medical devices, or contaminated environment in the same ward. Indeed, transient hand carriage of *C. difficile* spores by healthcare workers has been recognized as one of the main transmission routes for this pathogen [24, 25]. Of note, such a transmission should theoretically only be considered before diagnosis of the index case given the specific isolation measures proposed upon diagnosis. However, a post-infectious excretion of *C. difficile* might contribute to an ongoing transmission, as isolation measures are stopped 48 h after diarrhea resolution.

The presence of type B links (ward-to-patient) in four clusters suggests environmental contamination with *C. difficile* spores. This finding is of particular interest since sporicidal biocides are systematically used for disinfection of the environment of CDI patients in our institution and might suggest an insufficient effectiveness of these measures. Of note, while longer potential ward contamination periods have been previously used [26], we limited this period to 30 days to improve the robustness of the potential epidemiological links in accordance with previous studies [22, 23]. Interestingly, more than one potential epidemiological link was identified in some clusters, and, although type A link was considered the strongest, type B transmission could not always be excluded given the complex in-hospital patient trajectories in some cases (Fig. 3). A better exploration of the most plausible epidemiological link could be achieved through the evaluation of additional parameters, such as interpatient exposure, incubation period, and minimum ward contamination period, as previously reported [26].

The presence of only type C links was found in two clusters and no link in one cluster. While overlapping hospitalizations in different wards with different medical care teams have been previously described as the only epidemiological link between some CDI cases sharing the same genomic cluster [27], the underlying transmission routes, if any, remain to be elucidated. Possible explanations could be that these patients have shared briefly spaces that may have not been tracked by the epidemiological investigation, such as the emergency unit, the recovery room, or the radiology units, or were handled by a same healthcare worker poorly adherent to standard precautions and working in several departments. Cases of genomic clusters with poor or no epidemiological link could nevertheless be part of a nosocomial chain of transmission, which is consistent with current evidence that transmission between symptomatic inpatients accounts for only a part of CDI [7]. Several hypotheses might explain these acquisitions. Firstly, it is possible that some potential cases belonging to the genomic clusters remained unidentified, either because CDI or colonization remained undiagnosed or documented after hospital discharge. In fact, asymptomatic colonization, albeit potentially transient, may play an important role in the onward nosocomial transmission [28], as indicated by the presence of serum and colonic antibodies against *C. difficile* toxins in a significant part of the general population [29, 30]. In a study conducted by Curry et al., multilocus variable number of tandem repeats analysis showed that almost 30% of all isolates from HO-CDI were associated to isolates from asymptomatic carriers identified upon admission screening [31]. In another population-based prospective cohort study conducted in 2 university hospitals in Denmark, CDI was detected in 4.6% of patients exposed to asymptomatic carriers at the ward level compared to 2.6% of patients not exposed to carriers (OR 1.79; 95% CI: 1.16–2.76) [32]. In view of these results and the fact that 7.5–11% of hospitalized patients have been identified as colonized in previous studies [31, 33], this subpopulation could form a substantial reservoir for CDI given the large inpatient pool, especially in big institutions such as ours. Hence, current symptom-based infection control measures [34] may have only limited effectiveness for the prevention of nosocomial transmissions. In this context, universal screening and isolating carriers could be a preventive strategy, contributing to a significant decrease in incidence of HA-CDI [35], but its cost-effectiveness needs to be validated by further studies. Finally, some CDI cases diagnosed several days after hospital admission may represent late-onset CA-CDI and share a common community reservoir, where conventional risk factors are lacking [36], as it has been shown by genomic analyses in previous studies [8]. Indeed, a recent Swiss study showed that nearly 30% of inpatients with CDI were diagnosed within 72 h from admission suggesting common community acquisition [37].

Interestingly, in the largest cluster (A), strong epidemiological links (type A) were identified among all patients. Conversely, in smaller clusters, weaker (C, E, F, and G) or even no epidemiological links (C and D) were identified. This observation may suggest that transmissions occurring through weaker epidemiological links tend to be limited and less prolonged, as compared to those occurring through strong epidemiological links, which seem to spread to a broader extent and last longer.

Interestingly, all isolates of each genomic cluster belonged to the same DLST type, underlining the potential utility of DLST for the epidemiological surveillance of CDI cases. Thus, using DLST as first-line typing method followed by sequencing of only one isolate per patient and per DLST type shared with another patient (*n* = 60) is undoubtedly less costly than the universal sequencing of all isolates (*n* = 86). In fact, considering a cost per DLST of 20 euros per analysis and a cost of cgMLST of 205 euros per analysis, the first approach resulted in a saving cost of 3610 euros in our study, corresponding to a cost reduction of 20%. Hence, the use of DLST for the initial cluster analysis, followed by whole genome sequencing analysis for isolates sharing the same DLST, could be a promising and cost-effective stepwise approach for retrospective epidemiological investigations, although its processing time hinders the real-time detection of nosocomial outbreaks and implementation of infection control measures at the time being.

Our study presents a promising approach to describe the phylogenic relationships among *C. difficile* nosocomial strains using cgMLST and to correlate them with potential epidemiological links. However, it has several limitations. First, to evaluate the utility of this approach for the detection of clinically unidentified nosocomial transmissions, we retrospectively went through a limited period and used a relatively small sample size in a single institution, which does not allow the generalizability of the results. Second, we did not correlate the microbiological and epidemiological results with patients’ clinical characteristics, such as severity of symptoms and immunocompromised status, which may be associated with longer periods of colonization and prolonged contagiousness. Third, only spatiotemporal information was used for the epidemiological investigation, leaving metadata such as infection control interventions and antibiotic treatment outside the global analysis. Fourth, while cgMLST is an interesting tool to retrospectively identify nosocomial transmission chains, it is of limited utility for the early detection and the real-time management of hospital outbreaks due to the current complexity and turnaround time of this approach.

In conclusion, epidemiological surveillance of HA-CDI cases remains challenging. A stepwise approach including DLST and cgMLST analysis may be a promising, cost-effective approach for the genomic classification of *C. difficile* isolates, but more efficient epidemiological investigation strategies are still necessary to confirm transmission links among them. Prospective studies with larger samples and environmental testing could help address these unmet needs.

### Supplementary Information

Below is the link to the electronic supplementary material.

Supplementary file1 (PDF 219 KB)

Supplementary file2 (XLSX 14 KB)

## Data Availability

The authors confirm that all supporting data, code, and protocols have been provided within the article or through supplementary data files. Sequence read files have been deposited in the European Nucleotide Archive under the study number PRJEB56399.
