# Supplementary material for: Core genome multilocus sequence typing of Clostridioides difficile to investigate transmission in the hospital setting
Source: Eur J Clin Microbiol Infect Dis. 2023 Oct 23;42(12):1469–76. doi: 10.1007/s10096-023-04676-9 (PMC10651541; doi:10.1007/s10096-023-04676-9)
Supplement: Supplementary file 1 — Supplementary file1 (PDF 219 KB) [file 10096_2023_4676_MOESM1_ESM.pdf]

Core genome multi-locus sequence typing of *Clostridioides difficile* to investigate nosocomial transmissions

Supplementary material

Double locus sequence typing of *Clostridioides difficile*

The DLST scheme previously published (Stojanov et al. 2016) was modified as followed. A bacterial suspension was obtained by homogenizing a full 1µl loop collected on one isolated colony in 25 µl of sterile water. For one reaction, the PCR mix was prepared with 5.5 µl of GoTaq Green G2 master mix 2X (Promega, Madison, USA), 4 µl of H_2O and 0.5 µl of mix primers, each at an initial concentration of 12.5 µM. One microliter of the bacterial suspension was added to 10 µl of the mix PCR. PCR was performed with 5 min at 95°C followed by 28 cycles of 30 sec at 95°C, 30 sec at 63.5°C, and 30 sec at 72°C; followed by a final elongation of 2 min at 72°C.

Table . Primers used start trimming and sequence length for new DLST scheme of *C. difficile*

gene	graR	pglF
Primer For	actggaggagattttgctgaga	tagcgtactagtagcgtttcga
Primer Rev	agcttccaaagaatgtccacaa	gctctctrcccaacaartcttc
Start trimming sequence reverse	RTCTATTGCYACCATCATATT	--
Start trimming sequence forward	--	ATTTAYYACTAATGATATAATAATTTCAG
sequence length	639	664

Stojanov M, Magalhaes B, Terletsky V, Basset P, Prod'hom G, Greub G, Senn L, Blanc DS. Development and evaluation of double locus sequence typing for molecular epidemiological investigations of *Clostridium difficile*. Eur J Clin Microbiol Infect Dis (2016) 35:175–181

Blanc D.S., Poncet F., Grandbastien B., Prod'hom G., Greub G., Senn L Molecular typing of *Clostridioides difficile* from frozen stool samples to investigate cross-transmissions: A proof of concept. Indian journal of medical microbiology. 2022.

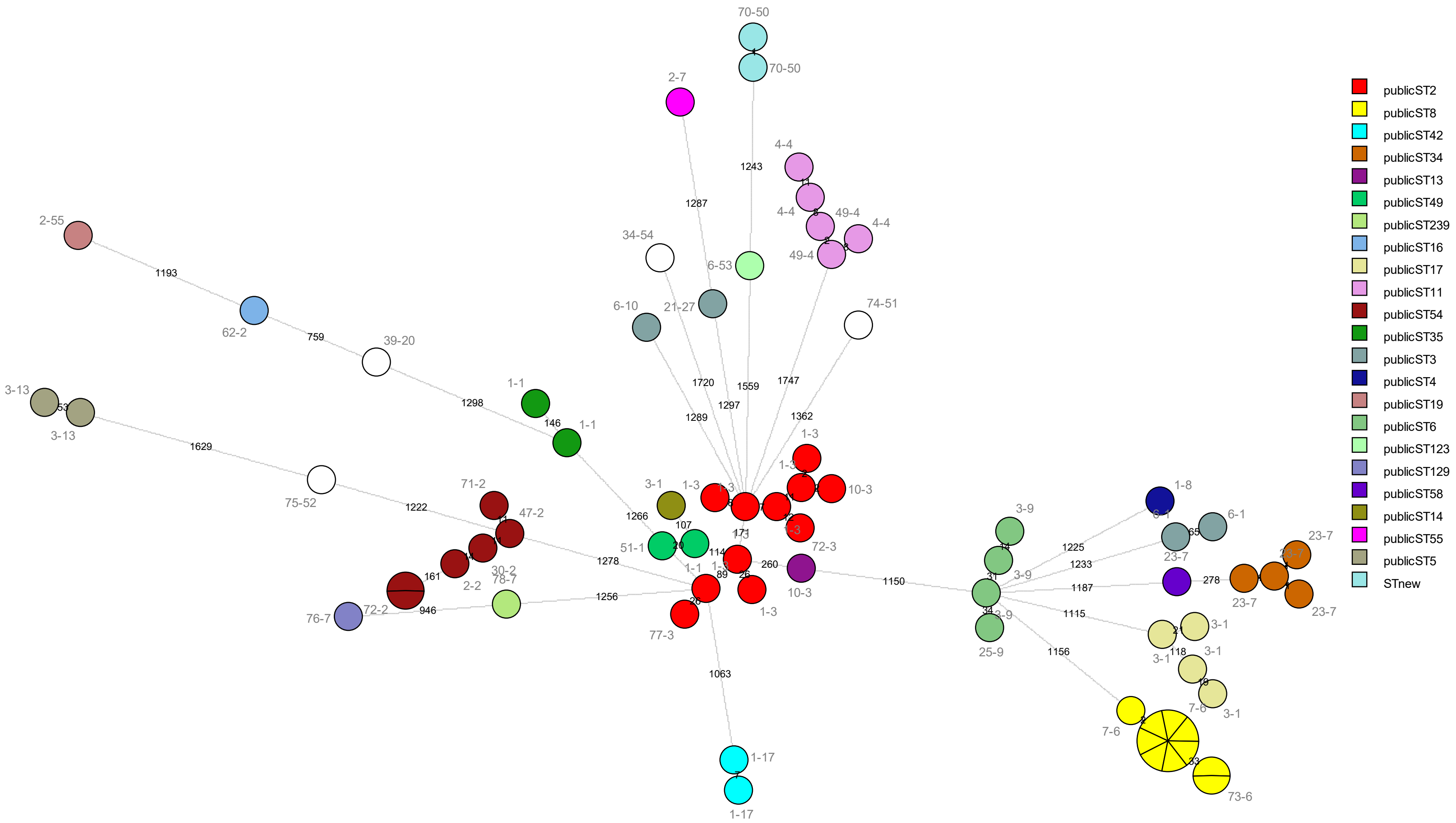

Figure S1. Minimum spanning tree with the 72 isolates based on cgMLST with DLST labels (FigureS1.svg)
